# Substantial fat mass loss reduces low-grade inflammation and induces positive alteration in cardiometabolic factors in normal-weight individuals

**DOI:** 10.1038/s41598-019-40107-6

**Published:** 2019-03-05

**Authors:** H. V. Sarin, J. H. Lee, M. Jauhiainen, A. Joensuu, K. Borodulin, S. Männistö, Z. Jin, J. D. Terwilliger, V. Isola, J. P. Ahtiainen, K. Häkkinen, K. Kristiansson, J. J. Hulmi, M. Perola

**Affiliations:** 1 Genomics and Biomarkers Unit, The Department of Public Health Solutions, National Institute for Health and Welfare, Helsinki, Finland; 2 Research Program for Clinical and Molecular Metabolism, Faculty of Medicine, University of Helsinki, Helsinki, Finland; 3 Sergievsky Center, Taub Institute and Departments of Epidemiology and Neurology, Columbia University, New York, NY USA; 4 Minerva Foundation Institute for Medical Research, Helsinki, Finland; 5 Public Health Evaluation and Projection Unit, The Department of Public Health Solutions, National Institute for Health and Welfare, Helsinki, Finland; 6 Public Health Promotion Unit, The Department of Public Health Solutions, National Institute for Health and Welfare, Helsinki, Finland; 7 Department of Biostatistics, Columbia University, New York, NY USA; 8 Departments of Psychiatry, Genetics & Development, Sergievsky Center, Columbia University, Division of Medical Genetics, New York State Psychiatric Institute, New York, NY USA; 9 Faculty of Sport and Health Sciences, Neuromuscular Research Center, University of Jyväskylä, Jyväskylä, Finland; 10 Department of Physiology, Faculty of Medicine, University of Helsinki, Helsinki, Finland

## Abstract

The accumulation of fat, especially in visceral sites, is a significant risk factor for several chronic diseases with altered cardiometabolic homeostasis. We studied how intensive long-term weight loss and subsequent weight regain affect physiological changes, by longitudinally interrogating the lipid metabolism and white blood cell transcriptomic markers in healthy, normal-weight individuals. The current study examined 42 healthy, young (age: 27.5 ± 4.0 years), normal-weight (body mass index, BMI: 23.4 ± 1.7 kg/m^2^) female athletes, of which 25 belong to the weight loss and regain group (diet group), and 17 to the control group. Participants were evaluated, and fasting blood samples were drawn at three time points: at baseline (PRE); at the end of the weight loss period (MID: 21.1 ± 3.1 weeks after PRE); and at the end of the weight regain period (POST: 18.4 ± 2.9 weeks after MID). Following the weight loss period, the diet group experienced a ~73% reduction (~0.69 kg) in visceral fat mass (false discovery rate, FDR < 2.0 × 10^−16^), accompanied by anti-atherogenic effects on transcriptomic markers, decreased low-grade inflammation (e.g., as α_1_–acid glycoprotein (FDR = 3.08 × 10^−13^) and hs-CRP (FDR = 2.44 × 10^−3^)), and an increase in functionally important anti-atherogenic high-density lipoprotein -associated metabolites (FDR < 0.05). This occurred even though these values were already at favorable levels in these participants, who follow a fitness-lifestyle compared to age- and BMI-matched females from the general population (n = 58). Following the weight regain period, most of the observed beneficial changes in visceral fat mass, and metabolomic and transcriptomic profiles dissipated. Overall, the beneficial anti-atherogenic effects of weight loss can be observed even in previously healthy, normal-weight individuals.

## Introduction

Visceral fat accumulation in the abdominal area has been shown to alter metabolite profiles, and is a significant risk factor for several chronic diseases^1–3^. Specifically, studies have reported that alterations in serum lipid levels and lipoprotein profiles, as well as levels of certain amino acids and inflammation biomarkers (e.g., triglyceride-rich lipoproteins (TRL), high-density lipoprotein (HDL)-cholesterol, α_1_-acid-glycoprotein, tumor necrosis factor alpha (TNF-α)) have been associated with the risk of cardiovascular disease (CVD), insulin resistance, type 2 diabetes (T2D), and metabolic syndrome^4–6^. Weight loss can, to some extent, ameliorate these adverse metabolic changes in individuals with relatively high levels of visceral fat^7^. Recent findings^8^ support the notion that reduction in visceral adiposity may be the primary mediator of the observed positive health effects, including reduction in serum triglyceride (TG) and low-density lipoprotein (LDL)-cholesterol levels, and increase in HDL-cholesterol levels. However, more detailed evidence is needed (i) to ascertain the specific role of visceral fat mass in biomarker modulation and (ii) whether comparable positive relations between visceral fat mass reduction and cardiometabolic profile exist even in normal-weight individuals.

In the 1940s, a seminal experiment investigated the effects of a long-term extreme low-calorie diet by subjecting young normal-weight males (n = 32) to a semi-starvation treatment for 24 weeks^9,10^. The study concluded that transient long-term semi-starvation (leading to weight reduction from 69.3 kg to 52.4 kg) does not cause any significant, long-term negative impacts on health after body weight is restored. For ethical reasons, subsequent weight loss studies have focused on the effects of severe weight loss only in overweight or obese individuals for a shorter duration (<10 weeks)^11–13^. These studies have repeatedly shown a range of beneficial health effects on biomarker profile and future disease incidence^14–17^. To date, however, our understanding of the health consequences of transient long-term weight loss in healthy normal-weight individuals, especially in females, is limited and highly warranted, due to high prevalence of weight loss attempts, even in the normal-weight general population. Potentially, a significant public health issue as over 50% of US females in their 20s to 50s have made efforts to rapidly lose weight in the past 12 months according to the US CDC (https://www.cdc.gov/nchs/data/databriefs/db313.pdf). To address the gap in current understanding, we aimed to determine: (i) whether reduction in body fat below normal levels would further improve cardiometabolic risk factors and (ii) how weight regain from low levels of fat mass affects the cardiometabolic profile and subsequent risk of CVD. Moreover, compared to overweight people, studying normal-weight individuals can reveal more about the physiology of human metabolism in its natural state, as obesity is considered to be a pathological, dysfunctional, and evolutionarily abnormal state.

Physique sports, which are judged on aesthetic appearance, require intense weight reduction by combining high volumes of aerobic exercise and resistance training with low energy intake. Athletes who participate in such activities are ideal study participants for assessing the physiological changes associated with weight loss in a population of normal-weight individuals. To explore the physiological changes associated with weight loss and regain, we integrated various omics approaches^18–20^ to examine the following biological entities sequentially: (i) *metabolomic serum products*, (ii) *enzymes that are involved in the regulation of plasma lipid metabolism*, and (iii) *transcriptomic profiles that may have contributed to variable expressions*. In addition, we examined the metabolomic profiles from individuals in an independent Finnish population-based study (FINRISK) to (i) confirm whether the findings from individuals competing in physique sports can be extended to the general population^21^, and (ii) explore how cardiometabolomic profiles are modulated in normal-weight individuals with similar Body Mass Indexes (BMIs) but different body compositions than physique athletes.

This study revealed that intensive weight loss and regain had striking effects on cardiometabolomic biomarkers and transcriptomic markers involved in anti-atherogenic and atherogenic pathways, respectively. These, in turn, may contribute to cardiovascular health in normal-weight individuals. Comparisons between age- and BMI-matched physique athletes and individuals from the general population revealed substantial differences in cardiometabolic profiles, thus highlighting the challenges of using BMI as an accurate predictor of cardiometabolic status.

## Results

### Overview

**Figure 1:**
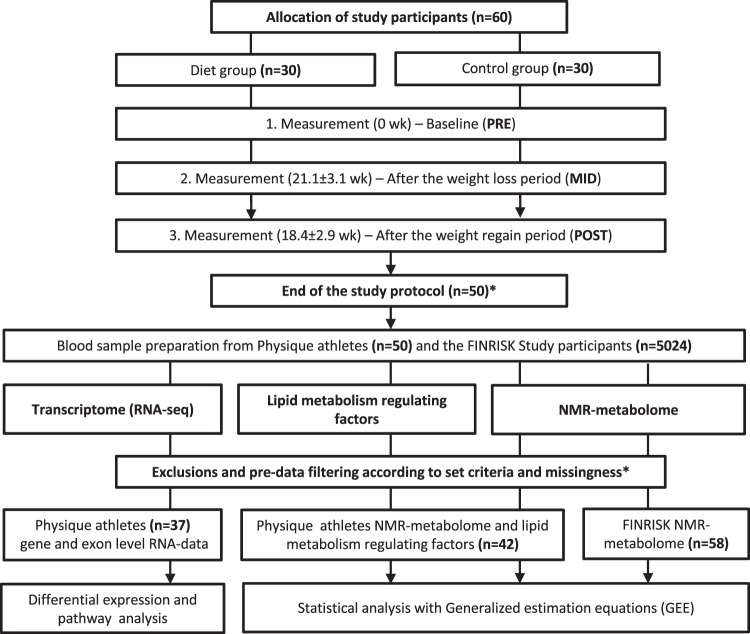
Study design and workflow. Flowchart illustrating the study protocol. The weight loss and regain period of the diet group are depicted in the upper section; the omics analysis protocol is depicted in the lower section. *The study began with 60 participants; 10 failed to complete the study regimen, one control did not arrive for baseline testing (PRE), and three dieters and six controls were excluded either because the duration of their weight regain period was shorter than the other participants, or because they failed to completely follow the instructions. Additional participants that lacked complete dietary records (n = 8) were excluded from the omics study due to the high cost of large-scale dataset quantification. Furthermore, sample size varied slightly between different downstream analyses due to incompleteness of omics or phenotype data.

Using an integrative omics approach, we investigated the metabolic and transcriptomic effects of intensive exercise and severe diet restriction period leading to weight loss, followed by subsequent weight regain (diet group; n = 25), and compared these profiles to those of non-dieting control individuals maintaining a roughly constant body weight (n = 17). In the diet group, the weight regain after intensive weight loss was voluntary, since caloric surplus and returning to “normal-weight” has been considered desirable when pursuing lean mass gains. As shown in Fig. 1, we evaluated the *metabolomic products*, *lipid metabolism-regulating enzymes/proteins*, and *transcriptomic markers* in these young (age: 27.5 ± 4.0 years) healthy, normal-weight (BMI: 23.4 ± 1.7 kg/m^2^) females. In addition, we examined metabolomic markers in a subset (n = 58) of young (age: 29.3 ± 2.5 years), healthy, normal-weight (BMI: 22.9 ± 2.6 kg/m^2^) females from an independent study (FINRISK^21,22^) derived from the Finnish general population. These individuals were age- and BMI-matched with the physique athletes, to explore the generalization of our findings in the physique athletes.

After the 20-week weight loss period, the diet group had achieved a reduction in fat mass across all measures (Table 1). Specifically, intensive weight loss in the diet group yielded a 73% reduction in visceral fat mass (false discovery rate, FDR < 2.0 × 10^−16^) in the abdominal area, which contributed to a 52% decrease in total body fat mass (FDR < 2.0 × 10^−16^) and an 8% reduction in waist circumference (FDR < 2.0 × 10^−16^) (Table 1). Weight loss was accomplished by a 19% decrease in energy intake and a 15% increase in total volume of exercise, as measured by relative energy expenditure (*metabolic equivalent hours per week*, METh/wk) (Table 1). In the controls, no meaningful changes were observed in the anthropometric traits measured throughout the study (PRE-POST) (Table 1).

**Table 1:** Characteristics of body composition, exercise level and dietary intake alterations in the Physique study groups and general population comparison FINRISK Study participants.

	Diet group (PRE)	Diet group (MID)	Diet group (POST)	Control group (PRE)	Control group (MID)	Control group (POST)	FINRISK
Weight (kg)	64.72 (6.92)	56.62 (5.51)*	63.17 (6.92)*	63.71 (5.07)	64.02 (5.76)	63.64 (5.55)	62.10 (7.84)
BMI (kg/m^2^)	23.54 (1.82)	20.60 (1.42)*	22.99 (2.03)*	23.08 (1.36)	23.20 (1.78)	23.05 (1.57)	22.90 (2.60)
Fat mass (kg)	14.88 (4.47)	7.17 (2.69)*	12.99 (4.21)*	14.19 (3.05)	14.87 (3.48)	14.39 (3.17)	17.80 (5.69)*
Lean mass (kg)	47.69 (4.2)	48.12 (4.03)	48.5 (4.43)*	47.52 (3.83)	47.44 (3.80)	47.53 (4.05)	44.30 (2.89)*
Waist circumference (cm)	75.66 (4.31)	69.58 (3.02)*	74.23 (3.92)*	74.18 (3.54)	74.0 (4.50)	72.90 (4.53)*	76.50 (7.23)
Waist:Hip -ratio	0.79 (0.03)	0.80 (0.04)	0.80 (0.03)	0.78 (0.03)	0.78 (0.03)	0.76 (0.03)*	0.81 (0.06)
Visceral fat mass (g)	937.92 (324.30)	249.56 (144.60)*	840.80 (306.80)	919.41 (327.70)	984.65 (379.41)	902.29 (350.30)	
Leg fat tissue thickness (cm)	0.98 (0.31)	0.64 (0.21)*	0.79 (0.28)*	0.97 (0.30)	1.02 (0.31)	1.08 (0.37)*	
Arm fat tissue thickness (cm)	0.94 (0.33)	0.69 (0.47)*	0.89 (0.36)	0.82 (0.23)	0.95 (0.24)*	0.94 (0.23)*	
Total exercise level (METh/wk)	59.30 (13.80)	68.40 (19.60)*	53.20 (16.20)	49.40 (27.80)	41.80 (18.70)	48.80 (27.00)	31.30 (19.70)*
Resistance training (METh/wk)	45.31 (8.76)	46.10 (9.90)	42.25 (8.23)	33.61 (19.44)	28.59 (14.22)	32.11 (17.77)	
Aerobic exercise (METh/wk)	13.95 (10.43)	22.3 (17.79)*	10.99 (12.19)	15.76 (23.81)	13.16 (14.54)	16.65 (25.61)	
Energy intake (kCal/kg)	36.51 (6.54)	29.62 (5.49)*	37.80 (9.87)	39.60 (8.04)	36.76 (5.77)	39.74 (5.46)	32.90 (10.20)*
Protein intake (g/kg)	3.14 (0.63)	3.06 (0.66)	3.34 (0.81)	2.77 (0.47)	2.80 (0.50)	2.86 (0.53)	1.43 (0.50)*
Carbohydrate intake (g/kg)	3.35 (1.02)	2.06 (0.64)*	3.24 (1.34)	3.58 (0.57)	3.42 (0.60)	3.59 (0.79)	3.99 (1.42)*
Fat intake (g/kg)	0.98 (0.25)	0.84 (0.22)*	1.02 (0.23)	1.28 (0.39)	1.17 (0.44)*	1.38 (0.46)	1.10 (0.35)

METh/wk = metabolic equilevant hours per week. kCal = kiloCalories. Values are presented as mean (standard deviation, SD). Means and SD’s are calculated for the physique athletes, n = 42. *Statistical significant diffence i) from baseline (p < 0.05) within Physique group comparisons and ii) between pooled Physique study participant baseline and FINRISK Study participants. Significance was calculated with Generalized Estimation Equations where age was accounted for in the model. Descriptives for general population comparison, the FINRISK Study cohort, was derived from the age- and BMI-(propensity score) matched individuals, n = 58.

### Weight loss: Overall metabolomic profile

**Figure 2:**
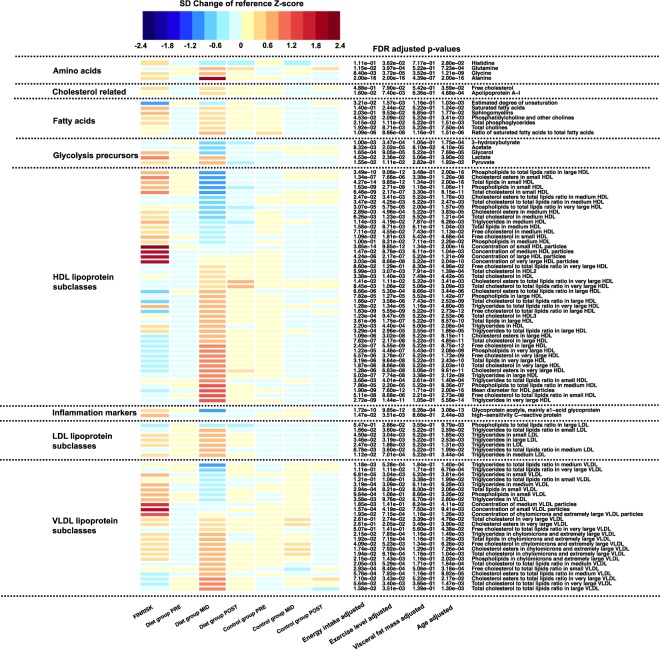
Heatmap of significant metabolite profile changes. Metabolite values and color key are represented as standard deviation (SD) change from reference Z-score. Calculated baseline Z-score values (PRE) from both diet and control group were pooled together and set as the reference level to which each individual group/timepoint-level was compared. FINRISK represents a subsample of age- and BMI-matched individuals from the general population (n = 58). On the heatmap, blue indicates decrease and red indicates increase in metabolite level compared to the calculated reference value. Multiple testing adjusted *P* values (false discovery rate, FDR) of the diet group analysis after weight loss (PRE-MID) are indicated in front of each metabolite name. Unadjusted basic model was defined as follows: metabolite ~ time + age. Factors known to contribute to metabolite levels were added as additional covariates to the basic model to determine their effect on observed modulation of metabolome profile. Energy intake, total exercise level, and visceral fat mass were accounted for in the model separately and are shown in the figure.

Using fasting blood samples, we examined metabolomic markers to determine whether intensive weight loss had significant effects on health-related biomarkers. Of the 230 metabolites that were screened, 100 had altered levels in the diet group, as measured by Nuclear Magnetic Resonance (NMR) spectroscopy and biomarker quantification (Supplementary Tables S1 and S2). As shown in Fig. 2, a dramatic weight loss in the diet group was most strikingly associated with beneficial changes in inflammation- and cardiometabolic-related biomarkers. In contrast, slight variation was detected in the metabolomes of the control group (Supplementary Table S3; Supplementary Results).

### Weight loss: Inflammation- and cardiometabolic-related biomarkers

Closer examination of the metabolomic profiles revealed decreased levels of systemic inflammation markers and increased levels of anti-inflammatory markers. Specifically, a decrease in an acute inflammation markers, high-sensitivity C-reactive protein (hs-CRP) (β = −0.24 ± 0.07, FDR = 2.44 × 10^−3^) and α_1_-acid glycoprotein (β = −0.16 ± 0.02, FDR = 3.08 × 10^−13^) were detected in the diet group (Fig. 2; Supplementary Table S2). Moreover, following weight loss in the diet group, total HDL-cholesterol (HDL-C) concentration substantially increased (β = 0.19 ± 0.04, FDR = 4.42 × 10^−5^), thereby resulting in cardiometabolically-beneficial modulation of this anti-inflammatory lipoprotein. Furthermore, we note that following weight loss, significant beneficial changes in size, number and composition of different HDL-subpopulations (n = 47) (FDR < 2.0 × 10^−16^) were observed, which have previously been associated with enhanced atheroprotective HDL functionality and reduced CVD risk^23–27^. Specifically, the weight loss period resulted in: (i) robust increase in large HDL-metabolites; (ii) decrease in small HDL-metabolites; (ii) increase in phospholipid content of large HDL-metabolites; (iv) increase in cholesterol and cholesterol ester content of large HDL-metabolites; and (v) increase in a major structural protein of HDL, apoA-I (β = 0.11 ± 0.03, FDR = 4.68 × 10^−4^) (Fig. 2; Supplementary Table S2).

### Weight loss: Lipid metabolism and triglyceride levels

A cardiometabolically-beneficial modulation of serum TGs distribution was observed, reflecting a further favorable alteration of lipid metabolism. Following weight loss, substantially lower levels of serum TGs in very-low-density lipoprotein (VLDL) were observed (β = 0.07 ± 0.03, FDR = 0.02) – a response that has been associated with reduced risk of CVD^28^ (Fig. 2; Supplementary Table S2). However, weight loss resulted in an increased level of TG content in HDL lipoproteins (β = 0.03 ± 0.007, FDR = 2.08 × 10^−4^). This modulation has previously been shown to be associated with improved function in phospholipid transfer protein (PLTP) reactions, whereby generation of larger HDLs and preβ-HDL particles ensures enhanced lipoprotein kinetics, cholesterol efflux from peripheral cells, and atheroprotection^29^. Alterations in the lipoprotein TG distribution did not result in changes in overall serum lipoprotein TG levels (within VLDL, IDL, LDL, HDL) following weight loss (FDR > 0.05) (Supplementary Table S2).

### Weight gain: Overall metabolomic profile

Next, we examined whether changes in metabolite levels following weight loss (PRE-MID) would be reversed following subsequent weight regain (MID-POST). By the end of the weight regain period in the diet group, levels of body weight and fat mass returned to the baseline levels (Table 1). Specifically, 98% of body weight, 87% of total body fat mass, and 90% visceral fat mass were gained back by the end of the study. Similar to the anthropometric measures, metabolite levels measured at the end of the weight regain period also approached the baseline levels (Fig. 2; Supplementary Table S4). Interestingly, 28 metabolites – comprising mainly HDL and different lipid metabolites – did not fully return to baseline levels (FDR < 0.05) (PRE-POST) and remained altered in a cardiometabolically-beneficial manner, supporting the possibility of a potentially sustained benefit (Supplementary Table S4).

### Regulating enzymes and proteins for lipid metabolism

To better understand the observed metabolomic changes associated with weight loss and regain mentioned above, we extended the metabolomic analysis to evaluate several enzymes and proteins that may explain the variation observed in the composition and size of the lipoproteins^30–34^. Consistent with the findings from our metabolomic analysis, we also detected significant alterations (FDR < 0.05) in some enzymes and proteins that regulate lipid metabolism – namely paraoxonase 1 (PON-I) and angiopoietin-like proteins 3 and 4 (ANGPTL3, ANGPTL4) – where time-dependent changes were observed in the diet group, but not in the control group (Supplementary Table S5; Supplementary Fig. S1).

Specifically, PON-1, an anti-inflammatory and -oxidative component of HDL, was up-regulated (β = 2.07 ± 0.89, FDR = 0.048) following the weight loss period in the diet group (Supplementary Fig. S1; Supplementary Table S5). We also detected a decrease in the level of ANGPTL3, an inhibitor of lipoprotein lipase (LPL) (β = −64.55 ± 22.32, FDR = 0.013), and an increase in PLTP (β = 362.29 ± 207.66, FDR = 0.14) that has previously been associated with an increased level of large HDL phospholipid content, consistent with our metabolomics findings (Fig. 2; Supplementary Table S5). Moreover, the level of ANGPTL4 – another LPL inhibitor, similar to ANGPTL3 – was reduced (β = −35.92 ± 10.22, FDR = 0.003), thus partially explaining the reduction in TG levels (VLDL-TG). Subsequently, circulating levels of all measured lipid-regulating enzymes and proteins returned close to the baseline levels by the end of the study, similar to the trend observed in the anthropometric measures and metabolomic markers (Supplementary Table S5). No other significant effects of weight loss or regain were observed in the lipid-regulating enzymes and proteins (Supplementary Fig. S1).

### Role of visceral fat mass in the alteration of lipid profile and low-grade inflammation

Following metabolomic marker analysis, we aimed to determine the underlying factors causing the widespread changes in the metabolomic profiles. In our Generalized Estimation Equation (GEE) models, accounting for visceral fat mass strikingly attenuated the observed time-dependent changes in the NMR-metabolome profile (Fig. 2; Supplementary Table S6). In contrast, other factors (e.g., exercise level, energy intake) had less significant effects when accounted for in the model (Fig. 2). As shown in Table 2, visceral fat mass was highly correlated with total fat mass (*r* = 0.80, *P* = 2.01 × 10^−6^) and arm adipose tissue thickness (*r* = 0.65, *P* = 8.10 × 10^−4^), but not as highly with leg adipose tissue thickness (*r* = 0.03, *P* = 8.71 × 10^−1^), representing lower body fat mass in the physique athletes. Our results suggest that reduction in upper body fat mass, especially visceral fat mass, has a relatively independent role in the modulation of metabolome lipid and inflammation-related biomarkers, most specifically on the pool of HDL subpopulations, subsequent to weight loss.

**Table 2:** Correlation coefficients and significance of different anthropometric measures, exercise level, and dietary information in relation to total fat mass in the Physique and FINRISK individuals.

	Diet group (PRE)		Diet group (MID)		Diet group (POST)		Control group (PRE)		Control group (MID)		Control group (POST)		FINRISK	
	*r*	*P* value	*r*	*P* value	*r*	*P* value	*r*	*P* value	*r*	*P* value	*r*	*P* value	*r*	*P* value
Weight (kg)	0.80	1.26E-06	0.71	8.23E-05	0.78	3.60E-06	0.62	8.26E-03	0.74	6.49E-04	0.68	2.52E-03	0.96	4.37E-32
BMI (kg/m^2^)	0.84	1.66E-07	0.70	9.16E-05	0.79	2.62E-06	0.56	1.84E-02	0.77	2.89E-04	0.68	2.56E-03	0.82	4.13E-15
Fat mass (kg)	Ref	Ref	Ref	Ref	Ref	Ref	Ref	Ref	Ref	Ref	Ref	Ref	Ref	Ref
Lean mass (kg)	0.20	3.43E-01	0.26	2.02E-01	0.21	3.04E-01	−0.03	9.00E-01	0.15	5.69E-01	0.09	7.42E-01	0.63	1.17E-07
Waist circumference (cm)	0.72	4.18E-05	0.39	5.09E-02	0.63	6.80E-04	0.61	8.83E-03	0.68	2.92E-03	0.78	1.98E-04	0.86	2.49E-18
Waist/Hip -ratio	−0.04	8.32E-01	−0.42	3.44E-02	−0.35	8.62E-02	0.25	3.28E-01	0.27	2.94E-01	0.55	2.08E-02	0.37	4.49E-03
Visceral fat mass (g)	0.92	1.42E-10	0.80	2.01E-06	0.92	1.14E-10	0.90	8.23E-07	0.92	1.54E-07	0.94	2.22E-08		
Leg fat tissue thickness (cm)	−0.09	6.79E-01	0.03	8.71E-01	−0.36	7.74E-02	−0.17	5.58E-01	0.04	8.80E-01	0.05	8.52E-01		
Arm fat tissue thickness (cm)	0.67	5.25E-04	0.65	8.10E-04	0.78	9.59E-06	0.46	9.76E-02	0.56	3.93E-02	0.70	4.92E-03		
Total exercise (METh/wk)	0.31	1.35E-01	−0.18	3.99E-01	0.37	1.53E-01	0.25	3.39E-01	0.29	2.54E-01	0.11	6.72E-01	−0.02	8.74E-01
Resistance training (METh/wk)	0.30	1.53E-01	0.17	4.36E-01	0.32	2.23E-01	0.05	8.57E-01	0.05	8.53E-01	0.37	1.47E-01		
Aerobic exercise (METh/wk)	0.16	4.49E-01	−0.29	1.67E-01	0.28	2.96E-01	0.25	3.34E-01	0.33	1.97E-01	−0.14	5.97E-01		
Energy intake (kCal/kg)	−0.14	5.15E-01	−0.33	1.15E-01	−0.6	8.11E-03	−0.21	4.25E-01	−0.23	3.66E-01	−0.27	3.38E-01	−0.38	3.35E-03
Protein intake (g/kg)	0.02	9.14E-01	−0.17	4.18E-01	−0.42	9.01E-02	−0.06	8.19E-01	0.04	8.83E-01	0.37	1.72E-01	−0.43	6.56E-04
Carbohydrate intake (g/kg)	−0.02	9.38E-01	−0.16	4.75E-01	−0.74	1.15E-03	−0.09	7.22E-01	−0.40	1.08E-01	−0.39	1.50E-01	−0.30	2.31E-02
Fat intake (g/kg)	−0.50	1.12E-02	−0.31	1.32E-01	−0.46	6.51E-02	−0.31	2.26E-01	−0.10	6.97E-01	−0.23	4.06E-01	−0.38	3.16E-03

Pearson correlation coefficients and significance were calculated from within group and time points in the Physique athletes (n = 42) and FINRISK participants (n = 58). Total fat mass was used as a reference measure (as in indicated by “Ref” in the table) to which other anthropometric measures, exercise levels, and dietary information was compared.

### Weight loss: Changes in the transcriptome

**Figure 3:**
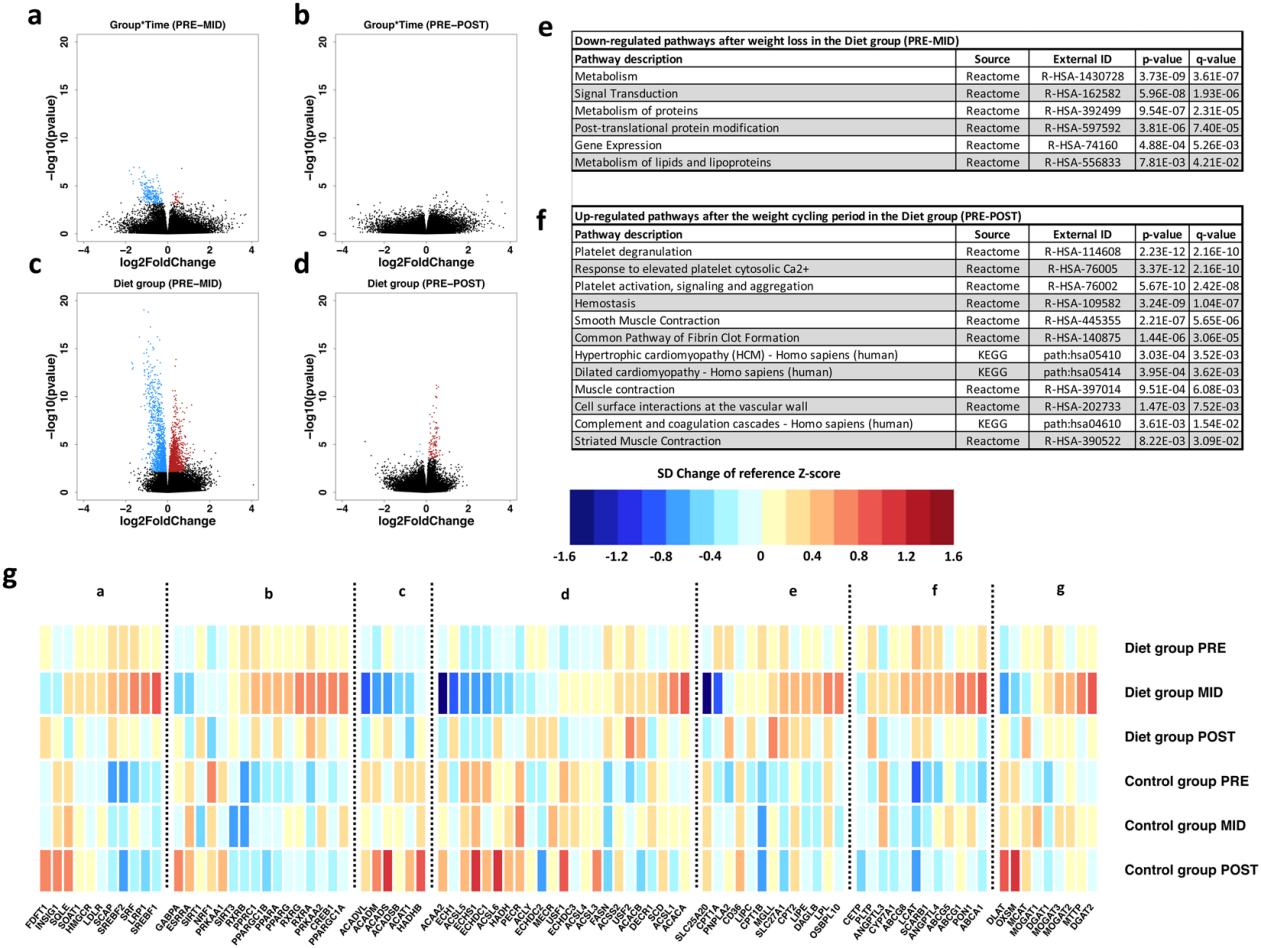
Volcano plots and most significant pathways of gene-level differential expression analysis results. Volcano plots in panels a and b represent Wald test contrast results from time-point interval comparisons between diet and control groups. Panels c and d depict results from diet group only analysis. Differentially expressed genes (DEGs) with statistically significant *P* values (false discovery rate, FDR < 0.05) (y-axis); blue indicates down-regulation and red indicates up-regulation. Magnitude of expression change is depicted on the x-axis with log2FoldChange. Panel e shows pathways associated with the 231 down-regulated DEGs from Time*Group interaction after the weight loss period (PRE-MID). No significant up-regulated pathways were found from the 24 up-regulated DEGs of the Time*Group interaction after the same period. Panel f depicts significant up-regulated pathways associated with 82 DEGs (panel d) affected by the whole weight cycling period in the diet group (PRE-POST) – although these genes were affected only by the weight regain period, not by weight loss. No significant down-regulated pathways were observed from the 5 down-regulated DEGs after the weight cycling period (PRE-POST). In panel g, lipid metabolism-related normalized gene expression levels and color key are represented as standard deviation (SD) change from reference Z-score. Baseline Z-score values (PRE) calculated from both diet and control group were pooled together and set as a reference level to which each individual group/timepoint level were compared; blue indicates decrease and red indicates increase in expression level compared to the reference value. a = Cholesterol synthesis, b = Energy metabolism regulation, c = Fatty acid oxidation, d = Fatty acid synthesis, e = Fatty acid mobilization and transport, f = HDL associated genes, and g = mono-, di-, triglyceride synthesis.

Finally, we examined changes in RNA expression levels in peripheral blood mononuclear cells (PBMCs) following weight loss and regain, using fasting blood samples. After the weight loss period, 255 differentially expressed genes (DEGs) were detected in the diet group when compared to the controls (FDR < 0.05) (Fig. 3; Supplementary Table S7). Of these post-weight loss DEGs, 231 were down-regulated and 24 were up-regulated in the diet group. The detected changes in RNA expression levels were rather minor (|log2FoldChange| < 2.0) and transient, as they returned to baseline levels after the weight regain period (Fig. 3). Post-hoc analysis of the diet group alone revealed a more widespread effect of weight loss on overall gene expression, as a total of 3,257 DEGs (FDR < 0.05) were detected (Supplementary Table S8). In addition, exon-level differential expression analysis supported the gene-level results, as the majority of the detected exons (44 exons at FDR < 0.01) belonged to the DEGs observed in the gene-level analysis (Supplementary Table S9).

In accordance with our metabolomic findings of the HDL profile and TG levels, DEGs after weight loss in the diet group were significantly associated (*q* value < 0.05) with the “Metabolism of lipids and lipoproteins” pathway (Fig. 3). Furthermore, closer inspection of the individual genes in this pathway revealed a significant up-regulation of genes associated with inhibition of hepatic lipogenesis (e.g., *OSBPL10*), TG-rich lipoprotein clearance (e.g., *LRP1, LPL*), HDL-mediated cholesterol efflux (e.g., *ABCA1, ABCG1, SCARB1*), regulation of TG metabolism in lipoproteins (e.g., *PPARGC1*), fatty acid transport to cells (e.g., *FATP*), fatty acid synthesis (e.g., *FASN*), and cellular cholesterol metabolism (e.g., *SREB-1, SREB-2*) (Fig. 3). Conversely, genes associated with fatty acid transport and oxidation in mitochondria (e.g., *SLC25A20, ACADS*) were down-regulated (Fig. 3).

### Weight cycling: Specific changes in gene expression and related pathways

Post-hoc analysis of the diet group alone revealed that the weight cycling period (PRE-POST) resulted in differential expression of 87 genes (FDR < 0.05) that were affected only by the weight regain period (Fig. 3; Supplementary Table S10). Of these 87 DEGs, 5 were down-regulated and 82 were up-regulated compared to the baseline (PRE-POST). Pathway analysis of the up-regulated DEGs revealed significant (*q* value < 0.05) associations with several pathways related to adverse cardiovascular processes and blood-related signals (e.g., hemostasis, platelet activation, signaling and aggregation, formation of fibrin clot, smooth muscle contraction, dilated cardiomyopathy, and hypertrophic cardiomyopathy) (Fig. 3).

### Comparisons with the general population: Weight loss and weight gain biomarkers

**Figure 4:**
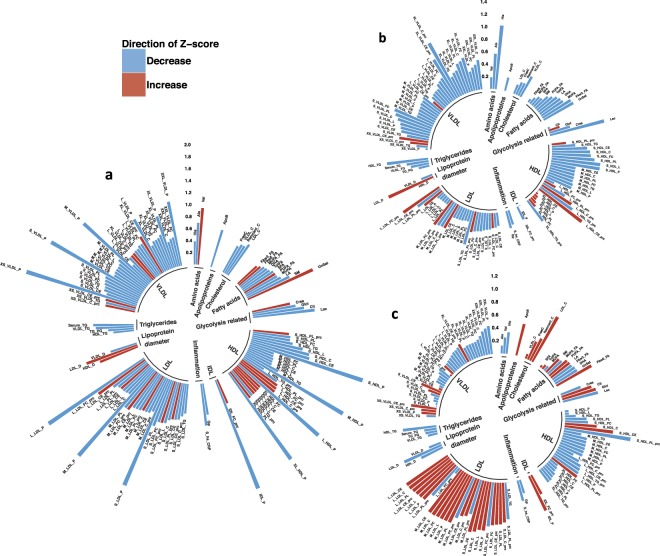
Polar bar plots of metabolome differences across physique and FINRISK participants. 129 health-related biomarkers that differed between the physique and FINRISK groups were plotted to demonstrate how general population metabolite profile (i) is altered compared to the physique athletes, and (ii) how metabolome profile is affected by weight gain and weight loss in this subsample of individuals from the general population. Polar plots are derived from metabolite raw-values (excluding outliers that were 4 standard deviation (SD) from the mean). Metabolite values are plotted as SD change from the reference Z-score. Red indicates increase and blue indicates decrease compared to the reference Z-score. Lipoprotein subclasses are further ordered according to size in a clockwise direction. Panel a. Physique athletes (diet and control group, n = 42) were pooled together at baseline to increase sample size when compared to age- and BMI-matched general population FINRISK individuals (n = 58). Metabolite values of FINRISK individuals were set as reference Z-score to which physique athletes were compared. A subset of these previously normal-weight FINRISK individuals who lost weight (n = 7, panel b) and gained weight (n = 13, panel c) during a 7-year follow-up was explored to determine if metabolome profile – including these 129 metabolites – was affected in similar manner as in the physique athletes after weight loss and weight gain. FINRISK individual baseline information (2007) was set as the reference Z-score to which the follow-up metabolome profile of 2014 was compared (panels b and c).

To determine the differences between individuals with years of training experience and a healthy lifestyle (e.g., physique athletes), and females from the general population of similar age and BMI, we compared the metabolomic profiles from all physique athletes (n = 42) at baseline with BMI- and age- matched individuals (n = 58) from the National FINRISK study (Table 1). This comparison revealed significant differences in the levels of 129 metabolites between the two groups (FDR < 0.05) (Fig. 4; Supplementary Table S11). We detected that lipid profiles, inflammation markers, and other health-related biomarkers (e.g., α_1_-acid glycoprotein, C-reactive protein, hs-CRP, serum total TGs and cholesterol, degree of unsaturation) were at a more favorable level in the physique athletes compared to the FINRISK individuals (Fig. 4; Supplementary Table S11).

Despite being matched in age and BMI, the two groups had distinct differences in body composition (FDR < 0.05). Specifically, the physique athletes had ~22% less fat mass, ~3% smaller waist:hip -ratio, and ~7% higher levels of lean mass when compared to matched FINRISK individuals (Table 1). However, this heterogeneity in body composition between the two groups only partially explained the differences in metabolomic markers: accounting for fat mass and lean mass dissipated only some of the differences observed in their metabolomic profiles (Supplementary Table S12). Consistent with our findings in the physique athletes, body composition differences better explained the differences in inflammation-related marker levels, but less so in the lipid profiles (Supplementary Tables S11 and S12). The physique athletes had ~43% higher physical activity (METh/wk), but this did not further explain the observed metabolomic profile differences (Table 1; Supplementary Tables S13). These observations suggest that other factors (e.g., other exercise-related features and healthy diet) associated with the lifestyle of the physique athletes might have a greater contribution to the detected differences, especially regarding lipid profiles.

Lastly, we explored how weight loss and regain affected the metabolomic profile in the subset of FINRISK individuals who had altered adiposity (n = 20) after a 7-year follow-up. Consistent with our findings from the physique athletes, fat mass loss of ~10% (n = 7) resulted in similar cardiometabolically-beneficial alteration in inflammation markers, serum cholesterol and lipid distribution, and lipoprotein composition (Fig. 4). Whereas, fat mass gain of ~20% (n = 13) induced adverse changes in cholesterol levels, lipoprotein distribution, and composition (Fig. 4).

## Discussion

The prevalence of weight loss attempts in modern society is increasing, even among individuals within a normal-weight range (https://www.cdc.gov/nchs/data/databriefs/db313.pdf). As such, insights on the possible health effects of weight loss and subsequent weight regain (e.g., weight cycling, transient weight loss) among a normal-weight population are greatly needed. We demonstrated for the first time that even in healthy, previously lean individuals with rather low quantities of fat mass, further reduction of body and visceral fat mass results in positive changes, mainly in anti-atherogenic lipid levels (e.g., VLDL-TG reduction), HDL profile (e.g., increase in HDL-C, apoA-I, HDL particle size, HDL particle number, and HDL phospholipids), and inflammation-related biomarkers (e.g., decrease in α_1_-acid glycoprotein acetyls and hs-CRP) (Fig. 2; Supplementary Tables S2). In our study, these cardiometabolically-advantageous changes to the lipid profile and inflammation-related biomarkers were mostly explained by changes in visceral fat mass (Fig. 2; Supplementary Table S6). However, the majority of the observed physiological changes reverted back to baseline levels during the weight regain period, which involved a decrease in exercise volume and increase in energy intake, resulting in baseline levels of body fat (Supplementary Table S2). Furthermore, compared with age- and BMI-matched females from the general population, females who maintain a body composition characterized by lower levels of fat mass have more favorable levels of several health-related biomarkers, including lipid levels (e.g., decrease in VLDL-TG and serum total TG), HDL profile (e.g., increase in HDL-C and HDL particle size, decrease in apoB:apoA-I ratio), and inflammation-related biomarkers (e.g., decrease in α_1_-acid glycoprotein acetyls and hs-CRP), thus demonstrating the benefits of maintaining lower levels of body fat (Fig. 4; Supplementary Table S11). Overall, our results suggest that i) further weight loss below normal levels of fat mass might have additional benefits in terms of cardiometabolic profile and future CVD risk, and that ii) lower levels of fat mass predicts more favorable levels of health-related biomarkers more accurately than BMI alone.

Obesity and visceral fat accumulation are characterized by adipose tissue dysfunction, relating to (i) chronic low-grade systemic inflammation (e.g., increase in α_1_-acid glycoprotein and hs-CRP) and (ii) adverse modulation of lipid profile (e.g., HDL-C decrease, TRLs increase), and (iii) increased risk of CVD^2,3^. Weight loss has been shown to alleviate these negative physiological changes in overweight individuals, thus attenuating the risk of CVD^35–37^. In accordance with our findings, it has been suggested that rather than changes in overall body weight, specifically the reduction of visceral fat mass and abdominal adiposity might better explain these improvements in lipid profile and low-grade inflammation^38^. Proportionally, a greater amount of visceral fat mass was lost when compared to the loss of total fat mass, which could partly explain our strong association between visceral fat mass change and metabolome profile response (Table 1). Earlier studies have shown that long-term aerobic exercise can alter serum HDL subpopulation profiles, independent of weight change^39^. To highlight and further determine the role of visceral fat mass in modulating the cardiometabolic profile, exercise and energy intake were accounted for in our analyses, as these factors are known to contribute to lipid metabolism (Fig. 2). In our study, however, compared to alteration in visceral and total fat mass, exercise level only explained a negligible amount of the observed differences in metabolome profiles i) after weight loss in the physique athletes and ii) between the age- and BMI-matched FINRISK individuals and physique athletes at baseline thus undermining the possibility for a major independent role of exercise in altering metabolome profile (Fig. 2; Supplementary Table S13). Nevertheless, exercise and energy intake are important factors in mediating weight loss, and their effects cannot be completely excluded. These findings affirm that (i) adiposity and fat mass distribution seem to be superior in predicting cardiometabolic profile and future CVD risk, compared to BMI or weight alone, (ii) visceral fat mass has an important role in influencing low-grade inflammation and lipid profiles, and (iii) the beneficial effects of exercise on the cardiometabolic profile are most probably mediated through inducing fat mass and visceral fat mass loss.

In the past, HDL-C has been proposed as one of the strongest epidemiological surrogates for protection against cardiovascular and coronary heart disease. Recently, human genetic Mendelian randomization and pharmacological studies aimed at increasing HDL-C levels have introduced controversy regarding the causality of this relationship^40–43^. HDL functionality is closely connected to the lipid/protein composition, quality (e.g., particle size) and molecular cargo associated with HDL particles^44^. In regard to HDL particle size, it has been shown that elevated levels of small HDL particles are correlated with increased risk of cardiovascular disease^45^, whereas larger HDL particle size reduces the risk^24^. Also, a negative correlation has been shown between HDL mean particle size and BMI, waist:hip ratio and serum TG levels, consistent with our findings^46^. In terms of CVD risk prediction, it has been suggested that HDL particle number/HDL-C might still be superior compared to HDL particle size^45^. In the present study, we observed a beneficial alteration in the overall HDL profile (e.g., increase in HDL-C, apoA-I, HDL particle size, HDL particle number, and HDL phospholipids), which was further supported by elevated LCAT and PLTP activity, decreased ANGPTL3 and ANGPTL4 levels and increased mRNA expression of *ABCA1*, *ABCG1, SCARB1* levels after weight loss whereas weight regain had the opposite effect (Figs 2, 3; Supplementary Fig. S1). In light of current knowledge, these alterations in HDL profile suggest enhanced atheroprotective functionality and reduced risk of CVD following weight loss. In support of these findings, we also detected similar modulations in HDL profile after weight loss and regain in the age- and BMI-matched females from the general population after a 7-year follow-up (Fig. 4).

Beneficial modulation of overall HDL profiles after weight loss in the physique athletes was accompanied by a potential adverse modulation of the HDL particle composition, as TG content of HDL lipoprotein increased (Fig. 2). Previous studies suggest that lipoprotein enrichment with TGs, observed in individuals with metabolic syndromes, might impair several lipoprotein functions i.e. interaction with receptors and plasma kinetics^47^, thus enhancing their atherogenic effect and increasing the risk of CVD. However, it has also been shown that TG-enriched HDLs can function as beneficial substrates for PLTP, generating large HDLs (and preβ-HDLs) that are associated with enhanced cholesterol efflux (e.g., from macrophage foam cells) and reduced risk of CVD^29^. Consistent with these findings, our observation of HDL profile (e.g., increase in HDL mean diameter and number of large HDL particles), lipid metabolism regulating proteins (e.g., increase in PLTP), cholesterol efflux activity (e.g., increase in *ABCA1*), and enhanced TG-rich lipoprotein clearance (e.g., increase in *LRP1* and *LPL*) suggest that weight loss-induced TG enrichment of HDL associates positively with enhanced reverse cholesterol efflux and atheroprotection (Figs 2 and 3). Additional studies including proteomics and detailed compositional analysis need to be pursued that examine the HDL particles generated during different physiological states (e.g., obesity, weight loss). This could more precisely determine the variance in HDL functionality and possible effects on cardiovascular health.

TG enrichment of HDL lipoproteins was not reflected on the total levels of lipoprotein TGs (VLDL, IDL, LDL, HDL), as they remained unaltered during weight loss. This result further attenuates the doubts of the possible adverse effects on HDL functionality and subsequent CVD risk (Supplementary Table S2). Previously, weight loss has been shown to reduce endogenous hepatic TG production, hepatic inflammation, and TG-enriched VLDL (VLDL-TG) secretion from the liver^48^. Consistent with these observations, VLDL-TG content, the major location of serum TG levels and subsequently a risk factor of CVD, was reduced as a result of weight loss in our study (Fig. 2). This was further supported by the up-regulation of *OSBPL10*, a gene responsible for coding the hepatic lipogenesis inhibiting protein, ORP10, which reduces VLDL-TG production from the liver^49,50^ (Fig. 3; Supplementary Table S7). In summary, inhibition of hepatic lipogenesis, together with reduced levels of VLDL-TG, and non-altered total levels of lipoprotein TGs imply overall cardiometabolically-favorable modulation of serum TG concentrations, distribution, and reduced risk of CVD after weight loss.

Weight gain is a significant risk factor affecting cardiometabolic homeostasis and increasing CVD risk, but it remains unclear whether weight loss followed by weight regain (i.e weight cycling) in previously normal-weight individuals can impact cardiometabolic health and CVD risk. Interestingly, we observed a notable up-regulation of genes associated with adverse cardiovascular outcomes and blood-derived signals after the weight cycling period (e.g., pathways related to the formation of fibrin clot, cardiomyopathy and cell surface interaction at vascular wall) (Fig. 3; Supplementary Table S10). These pathways were not affected by the preceding weight loss, but instead only by the weight regain – even when the body weight levels were not restored above the baseline levels (Table 1). Previous studies investigating gene expression in obesity and during periods of weight gain have reported similar results^17,51^. Overall, these results suggest that weight cycling-associated weight regain from low levels of body weight, regardless of i) preceding weight loss, ii) starting weight and iii) magnitude of weight regain, might have a negative impact on CVD risk and heart failure-related gene pathways.

Our study faced several limitations. Firstly, mRNA expression levels derived from leukocytes may not necessarily reflect actual levels of biologically-active proteins in tissues that are important for their physiological function. Secondly, despite a longitudinal design and thus strong statistical power as demonstrated by previous omics studies^17^, the sample sizes were relatively small. A similar study with larger sample sizes is warranted in order to validate the efficacy of our sample size and power to capture the biological variance in the measured variables. In spite of these challenges, our study had several strengths, most notably the comprehensive system biological datasets, longitudinal study design, control group, and a replicate cohort of age- and BMI-matched individuals from the general population.

In conclusion, significant weight loss leading to visceral fat mass reduction through high levels of exercise and energy restriction can further improve cardiometabolic profile through serum lipid levels, HDL profile, and inflammation-related biomarkers, even in previously normal-weight individuals. Our findings further highlight the enhanced capability of adiposity level and fat mass distribution in predicting cardiometabolic profile, compared to BMI or weight alone. More studies are needed to ascertain whether further beneficial alteration of cardiometabolic profile has any tangible implications in terms of CVD health. Similarly, further insight into the possible adverse cardiovascular outcomes related to induction of heart failure-related gene pathways after transient weight loss (i.e. weight cycling) could be beneficial for future recommendations for normal-weight individuals pursuing weight loss.

## Materials and Methods

### Study design and participants: the Physique study

The study cohort consisted of normal-weight (age: 27.5 ± 4.0 years, BMI: 23.4 ± 1.7 kg/m^2^) female physique athletes^19^. Participants volunteered to participate either in the diet (n = 30) or control group (n = 30) (Fig. 1). The participants in the control group were chosen through quasi-randomization by matching with those in the diet group based on age, BMI and the level of minimum training background indicated in the pre-study questionnaire.

As shown in Fig. 1, the physique athletes were measured at three time points: (i) baseline, prior to the weight loss regimen (PRE); (ii) after the diet period, which lasted 21.1 ± 3.1 weeks (MID); and (iii) after the weight regain period, which lasted 18.4 ± 2.9 weeks (POST). Participants in the diet group were engaged in rigorous exercise and lowered energy intake to ensure weight loss before the competition (PRE-MID). Following this period, they returned to normal levels of body weight and fat by increasing energy intake and reducing exercise level during the weight regain period (MID-POST). In contrast, participants in the control group were instructed to maintain their typical weight and usual fitness lifestyle, including regular exercise and healthy diet, and trying to maintain aesthetic body fat levels while increasing or maintaining muscle mass^19^ throughout the study period. At the three time points, participants in both groups went through a series of anthropometric and physical performance tests. A detailed description of the study design, participants, and methods was previously reported^19^. The Ethical Committee at the University of Jyväskylä approved the study protocol, and all participants gave written informed consent in accordance with the Declaration of Helsinki.

### Replication Study: the National FINRISK study

To determine whether the metabolomics findings from healthy normal-weight athletes also apply to the general population, we examined age- and BMI-(propensity score) matched females (age: 29.3 ± 2.5 years, BMI: 22.9 ± 2.6 kg/m^2^) from the DILGOM 2007 study (Dietary, Lifestyle and Genetic determinants of Obesity and Metabolic syndrome), which is a subsample of the National FINRISK study^21^. Of these individuals (n = 58), information from a 7-year follow-up (DILGOM 2014) was available for a subset (n = 20), allowing us to explore how long-term weight loss and weight gain alters the metabolic profile in this subset of young, normal-weight individuals from the general population. In order to ensure comparability with the physique athletes, several exlusion criteria were established: i) prevalent chronic disease, ii) prevalent lipid medication, and iii) pregnancy. For the study sample (n = 58), anthropometrics, physical activity level and dietary intakes are characterized in Table 1. The Ethics Committee of Helsinki and Uusimaa Hospital District approved the study protocols for the FINRISK study (decision number 229/E0/06) (DILGOM 2007) and DILGOM 2014. Participants provided signed informed consent in accordance with the Declaration of Helsinki.

### Anthropometric measurements

In the physique athletes, body composition and anthropometrics (including total fat mass, lean mass and visceral fat mass) were assessed with several methods, including Dual-energy X-ray absorptiometry (DEXA, Lunar Prodigy Advance, GE Medical Systems – Lunar, Madison, WI, USA) and B-mode axial plane ultrasound (model SSD-α10, Aloka, Tokyo, Japan). These methods were used to estimate subcutaneous fat tissue thickness of the arm (triceps brachii) and leg (vastus lateralis)^19^. For both physique athletes and FINRISK study cohorts, waist and hip circumference was measured using standard protocols: waist circumference was measured midway between the lower rib margin and iliac crest, and hip circumference was measured at the level of the widest circumference over the buttocks. For the FINRISK cohort, anthropometric measures of total fat mass and lean mass were taken using a bioimpedance machine (Tanita TBF-300MA, USA).

### Nutrient intake and physical activity

The physique athletes reported nutrient intakes repeatedly with dietary diary entries on representative days throughout the study: at baseline (**PRE**), after the weight loss period (**MID)**, and after the weight regain period (**POST**). Dietary information from the FINRISK cohort was collected using the Food Frequency Questionnaire taken during the baseline FINRISK study in 2007. For a more detailed description of nutrient intake information, see^19,21^.

For both study groups, the total physical activity level is similarly reported using metabolic equivalent hours per week (METh/wk) (Table 1). The physique athletes reported (i) type, (ii) duration, and (iii) intensity of daily physical activity throughout the study (PRE, MID, POST), from which overall physical activity (METh/wk) was calculated. Overall physical activity level for the FINSRISK cohort was derived from the International Physical Activity Questionnaire (IPAQ)^52^ taken during the FINRISK study in 2007.

### Blood samples

Fasting serum samples were collected from the physique athletes at three time points (PRE, MID, POST) for omics analyses. Blood was always drawn at the same time of day, following a fasting period of at least eight hours. Fasting serum samples were similarly obtained from the Finnish FINRISK cohort during the 2007 and 2014 collections.

### Metabolomics: NMR data preparation, quality control (QC), and management

A high-throughput serum Nuclear Magnetic Resonance (NMR) metabolomics platform was used for the absolute quantification of serum lipids and metabolites^53^. The full process and methods of sample preparation and quantification have been described elsewhere (http://www.computationalmedicine.fi/platform#method). The NMR metabolome assay yielded a total of 228 different metabolites, including an array of lipoprotein subclasses (e.g., VLDL, LDL, HDL), apolipoproteins (apo)A-1 and B-100, serum free fatty acids, and a wide variety of small molecules such as glycolysis precursors, amino acids and inflammation biomarkers (Supplementary Table S1). Fourteen lipoprotein subclasses, determined according to particle size, were analyzed as a part of the metabolite profile. In addition, calcium and high-sensitivity C-reactive proteins were quantified, resulting in a total panel of 230 different metabolites. We analyzed the metabolome of 42 physique athletes (diet group n = 25, control group n = 17) measured at three time points. The same NMR metabolite platform had been applied to serum samples from the FINRISK cohort (DILGOM 2007 and follow-up DILGOM 2014). In total, there were 229 common metabolites quantified in both populations that could be used for between-group comparisons (Supplementary Table S1). Only calcium was not available from the FINRISK cohort. Prior to analysis, we assessed data skewness, normality and outliers with dot plots and histograms. To dispose of excess variance caused by outliers, metabolite values were excluded from the analysis if standard deviation (SD) was greater or less than four (±4) from the mean.

### Measurements of enzymes and proteins that regulate lipoproteins

To determine the activities of enzymes and lipid transfer proteins that are important in regulating triglycerides (TGs), cholesterol and phospholipids balance between different lipoproteins, we studied phospholipid transfer protein (PLTP), paraoxonase (PON-1), cholesterol ester transfer protein (CETP), lecithin-cholesterol acyltransferase (LCAT), Angiopoietin-like (ANGPTL) 3, 4 and 8 proteins from serum samples of the physique athletes. Data skewness, normality and outliers were assessed similar to the metabolite values described above, and values with ±2 SD difference from the mean were excluded as outliers before statistical analysis.

CETP activities were analyzed with a radiometric method as a transfer/exchange of radiolabeled [14C] cholesteryl oleate (Amersham Biosciences) between exogenously added human LDL and HDL^30,54^. Radioactivity in HDL as a measure of transfer activity was determined by liquid scintillation counting. For the radiometric PLTP activity assay, radiolabeled phosphatidylcholine liposomes were prepared and the activity assay was carried out as previously described^31^. Prior to analysis, the fasting serum samples were diluted 1∶10 with assay buffer, and 4 µl of the dilution was used for the phospholipid transfer assay. After incubation, liposomes were precipitated and the radioactivity in HDL was measured by liquid scintillation counting. LCAT activities were measured with a radiometric method using radiolabeled reconstituted apoA-I-discoidal particles as a substrate, as described earlier in greater detail^32^. PON-1 activity was measured with a chromogenic method^33^. For these assays, intra- and inter-assay CVs ranged from 7% to 16%. In addition, plasma levels of ANGPTL3 and 4 were measured with ELISA methods, as described by Robciuc *et al*.^34^; ANGPTL8 was also measured with an ELISA method developed in our laboratory, as recently reported^55^.

### Statistical analysis of the metabolome and lipid metabolism factors

For statistical analysis of the metabolome and lipid metabolism-related factors, we used Generalized Estimating Equations (GEE) with linear link and working independence correlation structure.

To investigate whether levels of metabolites and lipid metabolism-related factors differed (i) between the diet and control group across any of the time points or (ii) between the physique and FINRISK cohorts, we applied GEE modeling accounting for between-subject variability and age. For the post-hoc analysis of the physique athletes’ metabolomics, the magnitude of change within diet and control groups was assessed across time points while accounting for between-subject variability and age as possible confounding factors.

In the physique athletes, the relationship between levels of metabolites and visceral fat mass change was tested by accounting for visceral fat mass in the GEE model described above. The visceral fat analysis aimed to determine if visceral fat mass reduction had an independent effect on the metabolome. Other factors known to contribute to lipid and metabolite profile (e.g., exercise levels, energy intake) were also accounted for. Similarly, the physique and FINRISK group comparison analysis further accounted for total lean, fat mass, and total physical activity to determine if (i) body composition or (ii) physical activity differences contributed significantly to the metabolomic profile despite matching based on age and BMI.

*P* value adjustment for multiple testing was carried out using Benjamini-Hochberg procedure (FDR) for all analyses conducted on NMR metabolome and lipid metabolism factors. All statistical analyses were carried out with R software (https://www.r-project.org).

### Transcriptomics: Library preparation, sequencing and read alignment

The RNA sequencing library for each sample was prepared using Illumina TruSeq following the manufacturer’s protocol (https://www.illumina.com). The Illumina protocol was paired-end, strand-specific, and the applied read depth for library preparation was set to 2 × 100 bp. All RNA > 200 bp was included in the prepared RNA-libraries whether they had a PolyA-tail or not. Ribosomal RNA was excluded from white blood cell samples accordingly with the ribodepletion method. Sequencing of the RNA libraries was performed on the Illumina Hiseq2000 platform.

No technical replicates were included in our dataset and no ERCC Spike-Ins were used as technical controls for the management of possible batch effect. The possibility of batch effects is likely to be minimal, since all samples were prepared by the same person using the same library manufacturing and sequencing methods. However, batch information was included in the statistical analysis as time-point information.

### Differential expression of gene-level data

We further processed sequence alignments with the DESeq2 software (http://bioconductor.org/packages/DESeq2) to assemble transcripts, quantify the expression levels and analyze differentially expressed genes (DEGs). Before statistical analysis, genes with very low expression were excluded, resulting in a total of 111 samples from 37 participants (diet group n = 24, control group n = 13).

For the detection of DEGs, we used a Likelihood ratio test to conduct a nested time-course study with DESeq2 (H_0_ = Group + Time + Group*Subject, H_1_ = Group + Time + Group*Subject + Group*Time), where we investigated if genes were differentially expressed between the diet and control group across any of the time points while accounting for the between-subject variability. We also conducted post-hoc analysis of the Likelihood ratio test for the diet and control group only (H_0_ = Subject, H_1_ = Subject + Time) to further explore within-group changes. In addition, we applied a Wald test within the DESeq2 interface for testing contrasts and deriving specific log2FoldChanges and *P* values for the between/within group comparison across any two individual time points (PRE-MID, PRE-POST). We used FDR to adjust *P* values for multiple testing; significance after adjustment was *P* ≤ 0.05.

### Differential expression analysis of exon-level data

In addition to gene-level tests, we conducted differential expression analysis at the exon level using the Bioconductor packages edgeR and Limma^56,57^. EgdeR was mainly used to prepare the data object before further filtering and normalization. Lowly-expressed exons were excluded based on two criteria i) if an exon was not expressed in any of the sample libraries, and ii) if CPM (counts per million) were <1 in less than three sample libraries. EdgeR implemented TMM data normalization before differential expression analysis. We conducted voom-transformation and differential expression analysis according to the Limma vignette, where we applied linear regression and empirical Bayes statistics to determine differentially expressed exons.

### Pathway analysis of gene-level data

Downstream pathway analysis was conducted to identify i) enriched and ii) over-represented biological pathways. The Web-based tool ConsensusPathDB-human (CPDB) database (http://cpdb.molgen.mpg.de) was used for analysis, as it combines a wide set of integrated databases. Enrichment and over-representation analysis focused on determining pathways from Reactome and Kyoto Encyclopedia of Genes and Genomes (KEGG) databases.

To calculate enriched pathways, we supplied a pre-ranked list of DEGs to the database engine. This DEG list was ranked based on −log10(*P* value) * sign(log2FoldChange) to account for both significance of differential expression and magnitude of expression change between groups across time points. Analyses were calculated separately for lists of up- and down-regulated DEGs to increase power. Only DEGs were used in this analysis to exclude redundant genes and to focus on affected gene pathways. The minimum number of genes enriched and over-represented in each pathway was >4 and >2, respectively, and significance was set at *q* value < 0.05.

### Code Availability

The bioinformatics scripts/codes generated for the statistical analyses are available upon request from the corresponding author.

## Supplementary information

Supplementary Dataset 2

Supplementary Dataset 1

## Data Availability

The datasets generated and/or analysed for the current study are not available to third-party individuals. In order to gain access to these datasets, applications must be submitted to the National Institute for Health Welfare, Helsinki, Finland, according to the terms of data distribution protocols set by the National Institute for Health Welfare, Helsinki, Finland.
