# Supplementary material for: Substantial fat mass loss reduces low-grade inflammation and induces positive alteration in cardiometabolic factors in normal-weight individuals
Source: Sci Rep. 2019 Mar 5;9:3450. doi: 10.1038/s41598-019-40107-6 (PMC6400952; doi:10.1038/s41598-019-40107-6)
Supplement: Supplementary file 1 — Supplementary Dataset 2 [file 41598_2019_40107_MOESM1_ESM.pdf]

Substantial fat mass loss reduces low-grade inflammation and induces positive alteration in cardiometabolic factors in normal-weight individuals

Sarin HV [1,2], Lee JH [3], Jauhiainen M [1,4], Joensuu A [1,2], Borodulin K [5], Männistö S [6], Jin Z [7], Terwilliger JD [1,8], Isola V [9], Ahtiainen JP [9], Häkkinen K [9], Kristiansson K [1,2] Hulmi JJ [9,10], Perola M [1,2]

1 Genomics and Biomarkers Unit, The Department of Public Health Solutions, National Institute for Health and Welfare, Helsinki, Finland
2 Research Program for Clinical and Molecular Metabolism, Faculty of Medicine, University of Helsinki, Helsinki, Finland
3 Sergievsky Center, Taub Institute and Departments of Epidemiology and Neurology, Columbia University, New York, NY, USA
4 Minerva Foundation Institute for Medical Research, Helsinki, Finland
5 Public Health Evaluation and Projection Unit, The Department of Public Health Solutions, National Institute for Health and Welfare, Helsinki, Finland
6 Public Health Promotion Unit, The Department of Public Health Solutions, National Institute for Health and Welfare, Helsinki, Finland
7 Department of Biostatistics, Columbia University, New York, NY, USA
8 Departments of Psychiatry, Genetics & Development, Sergievsky Center, Columbia University; Division of Medical Genetics, New York State Psychiatric Institute, New York, NY, USA
9 Faculty of Sport and Health Sciences, Neuromuscular Research Center, University of Jyväskylä, Jyväskylä, Finland
10 Department of Physiology, Faculty of Medicine, University of Helsinki, Helsinki, Finland

Supplementary results

Seasonal variation of body composition and metabolome profile in the control group

Similar large changes in the metabolite concentrations were not observed in the control group (Fig. 2). Despite being instructed to maintain invariable lifestyle and body weight, the control group showed cardiometabolically adverse trends (FDR < 0.05) that were indicated in HDL-related parameters during the first half of the study (PRE-MID) which was accompanied by a small ~5% increase in total fat mass (DEXA) (Table 1). Specifically, reduction in the levels of 13 different HDL-metabolites (FDR < 0.05) was observed after the MID time point measurements (Supplementary Table S14.) but all of these changes dissipated by the end of the study (POST). No significant long-term seasonal changes were detected within control group after study protocol when comparing to baseline values (PRE-POST) (Supplementary Table S3).

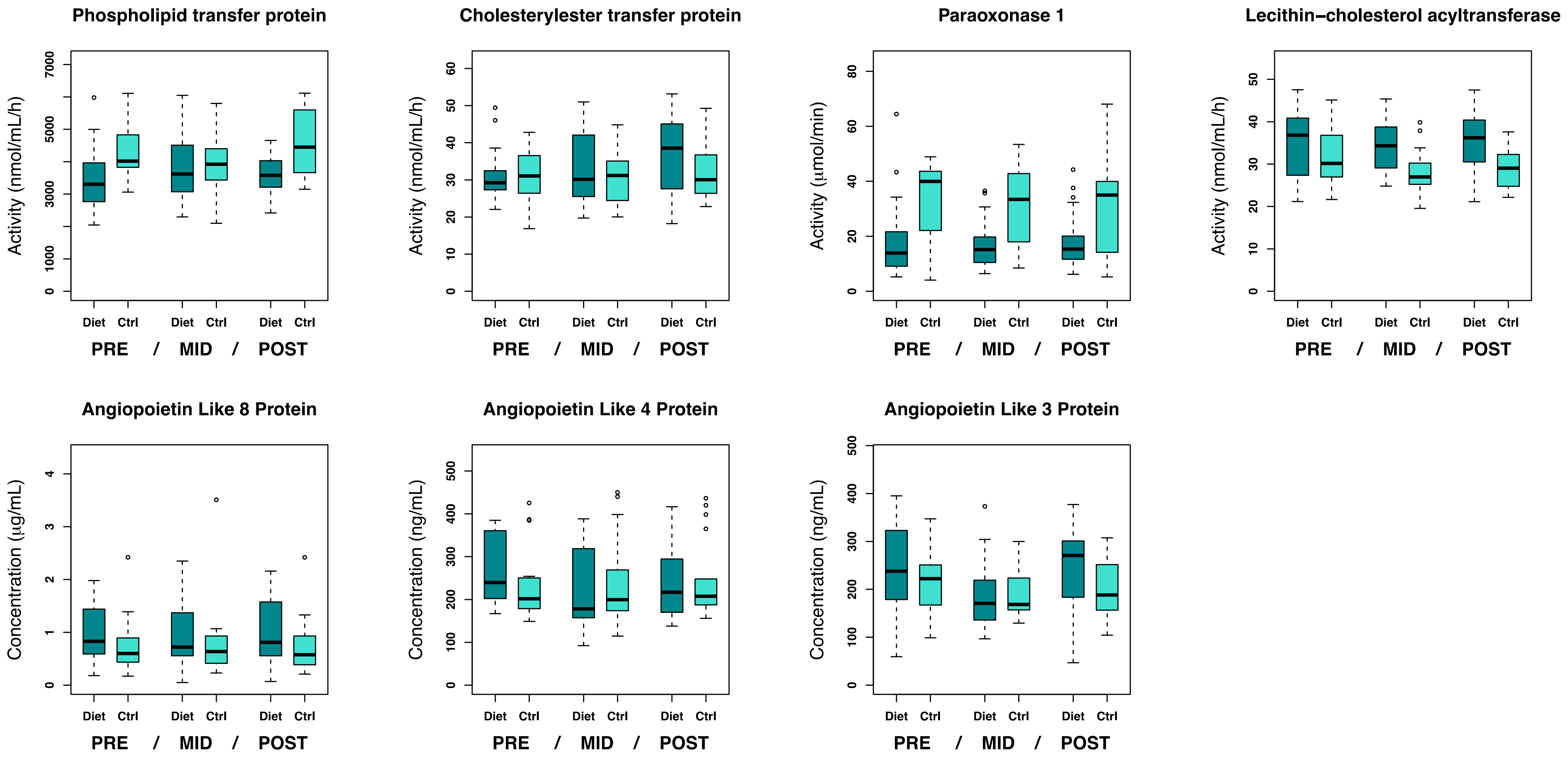

Supplementary Figure S1. Box-plots of lipid metabolism-regulating factors. Plots are derived from raw-values where outliers based on 2 standard deviation from mean have been excluded. Phospholipid transfer protein = PLTP. Cholesteryl ester transfer protein = CETP. Paraoxonase 1 = PON-1. Lecithin-cholesterol acyltransferase = LCAT. Angiopoietin Like 8 Protein = ANGPTL8. Angiopoietin Like 4 Protein = ANGPTL4. Angiopoietin Like 3 Protein = ANGPTL3.
